# Patient Perspectives Regarding Healthcare Professional Attire

**DOI:** 10.7759/cureus.57157

**Published:** 2024-03-29

**Authors:** Adriel Seah, Kaiwen Ng, Tony Ang, Sean Wei Loong Ho

**Affiliations:** 1 Orthopaedic Surgery, Tan Tock Seng Hospital, Singapore, SGP

**Keywords:** office, formal, scrubs, patient experience, doctors, orthopaedic surgery, professionalism, attire

## Abstract

Introduction

Physician attire has been shown to influence patients' impression of their healthcare provider. Scrubs and formal office attire are interchangeably worn by physicians. This study aimed to determine differences in scrubs and formal office attire on patient perceptions of professionalism, friendliness, aptitude, and empathy.

Methods

A single-center questionnaire study was conducted and a total of 150 patients were included. Patients were recruited from the inpatient and outpatient settings. Patients completed a questionnaire in which they rated 22 photographs. The photographs comprised a series of randomly arranged vignettes, with each participating physician appearing twice - once in formal office attire, once in scrubs. The physicians served as their own controls. Patients were asked to rate the depicted physicians based on the following four criteria - professionalism, friendliness, aptitude, and empathy. Each criterion was rated on an 11-point scale (0-10). Comprehensive demographic information, including age, gender, and race, was collected.

Results

A total of 150 responses were collected (50 inpatient and 50 orthopaedic surgery outpatient, 50 general medicine outpatient). Scrubs were rated significantly higher than formal office attire in all domains: professionalism [mean 7.52 (SD 1.95) vs. 6.69 (SD 2.38), p< 0.001], friendliness [mean 7.54 (SD 1.86) vs. 6.87 (SD 2.23), p< 0.001], aptitude [mean 7.44 (SD 1.99) vs. 6.72 (SD 2.36), p < 0.001] and empathy [mean 7.36 (SD 2.01) vs. 6.71 (SD 2.36), p < 0.001]. The perceived age of the physician did not affect any of the domain scores. Female physicians scored poorer in professionalism [mean 6.95 (SD 2.30) vs. 7.20 (SD 2.16), p < 0.05] compared to male physicians, but this difference resolved when analyzing only physicians wearing Scrubs.

Conclusion

Patients view physicians in scrubs as having higher professionalism, friendliness, aptitude, and empathy as compared to physicians in formal office attire. Physicians should don standardized colored scrubs with a prominent name tag to improve patient perceptions.

## Introduction

In today’s dynamic healthcare landscape, several components of care contribute to patient satisfaction [1]. Patient satisfaction has been shown to have a significant positive correlation with reduced 30-day re-admission rates, and even mortality [2-4]. As such, patient experience remains a key outcome measure of healthcare systems. In addition to clinical expertise and acumen, several other components of healthcare delivery may improve the patient experience [1]. Published literature has highlighted the importance of factors such as the waiting time, clinical environment, duration of consultation, and effective physician communication [1,5]. Healthcare professional attire has also been noted to be of importance to patients [6].

Patients often form immediate and lasting first impressions when meeting their healthcare providers. Such first impressions can be based on subtle cues that each healthcare provider exhibits, such as eye contact [7], gestures [7], and attire [6]. The clothing choices of healthcare providers can convey a sense of professionalism, friendliness, empathy, and aptitude. Modern clothing choices for physicians include white coats, formal office attire, and scrubs. Whilst it may have been more common previously for all physicians to don white coats, there has been a progressive move towards formal office attire and/or scrubs as the preferred attire. Infection control protocols have also reduced the desire for long-sleeve coats to reduce fomite transmission [8]. Apart from infection control measures, there have also been several studies that have investigated patients’ preferences for healthcare provider attire [9,10]. It has become clear that physician attire can influence patient perception [11]. However, it is important to recognize that geographical variation and a different cultural milieu among the studies may limit the generalizability of such data.

The authors hypothesized that patients’ perceptions of physicians would be similar between formal office attire and scrubs. This study aimed to determine patient perceptions of professionalism, friendliness, aptitude, and empathy of physicians in both formal office attire and scrubs.

## Materials and methods

A prospective, questionnaire-based study was performed in a single tertiary center in Singapore. Ethics approval was obtained for this study [2022/00923]. Patients were recruited from both the inpatient and outpatient settings. The inclusion criteria included all patients aged 18 to 90 years old who sought medical treatment in either the inpatient or outpatient setting. The following patients were excluded: Patients with vision or cognitive problems that did not allow them to review photographs and patients with working experience in the medical field.

Questionnaire design

A face-to-face paper questionnaire was administered by members of the study team. Participation was voluntary and anonymous, with no incentive for patients. Patients were recruited equally from three sites the orthopedic surgery outpatient clinic, the general medicine outpatient clinic, and the inpatient wards. The paper questionnaire was designed to be completed within 10 minutes to avoid fatigue. The following demographic data was collected: age (in years), gender (male/female), and Race (Chinese/Malay/Indian/others). No identifying factors were otherwise collected.

There were two parts to the questionnaire. The first section was designed to determine how patients differentiated between physicians and allied health workers, as well as to determine the perceived importance of attire and age of physicians.

Qualitative data collected was collected in the first section of the questionnaire:

1) How does the patient tell the difference between a nurse and a doctor?

Options offered were: a) the color of their uniform, b) the way they speak, c) their name tags, d) others.

2) Does the attire of the doctor affect the patient’s perception of their work?

Patients were tasked to answer with either ‘Yes’ or ‘No’, and asked to elaborate on their answer.

3) Does the age of the doctor affect the patient’s perception of their work?

Patients were tasked to answer with either ‘Yes’ or ‘No’, and asked to elaborate on their answer.

**Figure 1:**
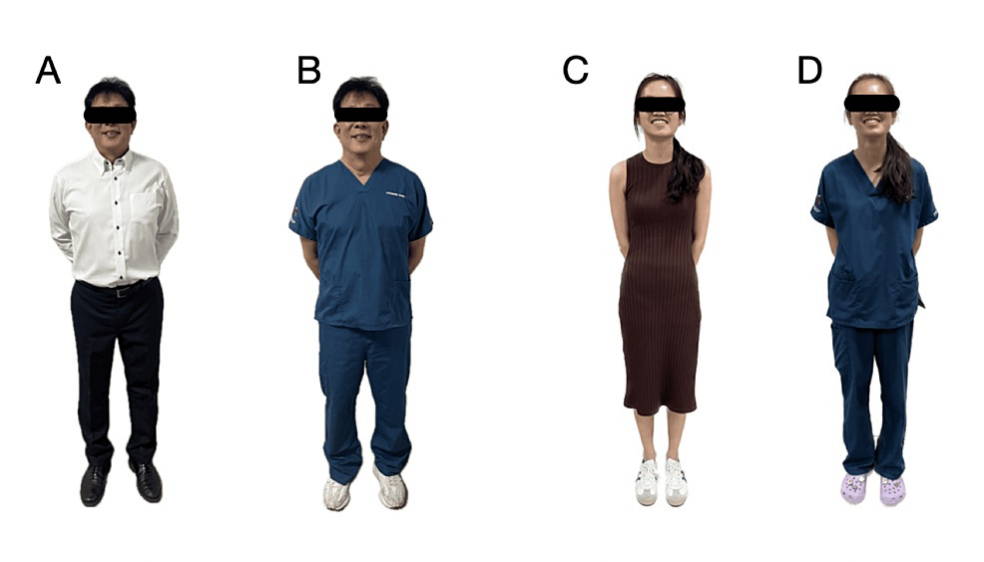
A - Male Physician 1 in Formal Office Attire. B - Male physician 1 in Scrubs. C - Female physician 2 in Formal Office Attire. D - Female physician 2 in Scrubs

The second section of the questionnaire was designed to elicit preferences regarding the two studied attires - formal office attire and scrubs. Photographs of 11 volunteers (including a mix of doctors and non-doctors) were provided. All 11 volunteers adopted a similar posture with a neutral facial expression. All photographs were lightly anonymized to reduce potential identifying features and potential bias. Each of the 11 volunteers had photographs taken of them in both the formal office attire and scrubs. The scrubs attire was standardized. Each volunteer served as their control, being dressed in both attires (Figure 1).

The volunteers were sourced and dichotomized further into the following subgroups: age (young vs old) and gender (male vs female). In view of the demographics within the local Singaporean public healthcare system, ‘older’ physicians were defined as physicians above the age of 40. This allowed for further subgroup analysis of patient preference.

Patients were tasked to rate each photograph based on an 11-point scale (0-10), based on their perceived ‘professionalism’, ‘friendliness’, ‘aptitude’, and ‘empathy’. The definitions of each of these domains were provided to the patients.

Statistical analysis

Descriptive statistical analysis was performed with frequencies and percentages for discrete data. Continuous data was analyzed with means and standard deviation or median with interquartile ranges. The differences between the scores of professionalism, friendliness, aptitude, and empathy were statistically analyzed using the student t-test. Categorical data was analyzed using the chi-square test where appropriate. A two-tailed significance level of 0.05 was used for all the tests. All statistical analysis was performed using STATA 13 (StatCorp, College Station, TX, USA).

## Results

A total of 150 questionnaires were completed by patients across the three clinical settings (orthopedic surgery outpatients, general medicine outpatients, and inpatient wards). An equal number of patients from each of these three settings were recruited (n = 50). A total of 148 completed questionnaires were analysed (two incomplete questionnaires). There were a total of 72 males (49%) and 73 females (49%), with three patients declining to answer. The median age was 55 years (SD 17.1). There were 77 (52%) young patients (aged < 60 years) and 71 (48%) old patients (aged ≥ 60 years). Other demographic data is presented in Table 1.

**Table 1:** Patient Demographics

	Respondents (n=148)
Mean Age (Years)	55 (SD 17.1)
Young Patients (< 60 years)	77 (52%)
Old Patients (≥ 60 years)	71 (48%)
Gender	
Male	72 (49%)
Female	73 (49%)
Declined to answer	3 (2%)
Race	
Chinese	101 (68%)
Malay	18 (12%)
Indian	8 (5%)
Others	5 (3%)
Declined to answer	16 (11%)

How do patients identify doctors from nurses?

Patients most commonly identified doctors from nurses via the color of their uniform (n=111/148, 75%). The other ways included name tags (n=43/148, 29%) and manner of speech (n=19/148, 13%). Patients also recognized doctors by the donning of stethoscopes (n =5/148, 3%). In their responses, patients elaborated that oftentimes they recognized the doctors’ scrub's color from the nurse's scrub/uniform color but were unable to elaborate on which colors they associated with doctors. When asked to elaborate on how speech allowed patients to recognize the difference between a doctor and a nurse, the majority of patients explained that a more ‘formal’ and ‘professional’ speech indicated that someone was a doctor as opposed to a nurse (Table 2).

**Table 2:** Differentiating between physicians and non-physician Percentages do not add up to 100% as patients were allowed to select multiple choices.

	Respondents (n=148)
Color of Scrubs	111 (75%)
Name Tags	43 (29%)
Manner of Speech	19 (13%)
Donning of Stethoscope	5 (3%)

Physician attire and perceived professionalism

In this first section of the survey, patients had not yet been tasked to review the photographs of doctors in their different attires. We had asked them if the attire of the doctor would have affected how patients viewed their professionalism. Notably, the large majority of patients indicated that the attire of a doctor had no bearing on their perceived professionalism [98/148, 66% (no bearing) vs 51/148, 34%]. 

Age of a doctor and perceived professionalism

Patients were asked if the perceived age of their doctor would affect their perceived professionalism. Patients largely believed that the perceived age did not affect how they viewed the doctor’s professionalism (111/148, 75% vs 36/145, 24%).

Patient ratings of formal office attire vs. scrubs

Patients rated the scrubs significantly more favorably as compared to the formal office attire in all domains measured (Table 3).

**Table 3:** Domain scores between Scrubs vs. Formal Office Attire

Domain	Scrubs Mean (SD)	Formal Office Attire Mean (SD)	P-Value
Professionalism	7.52 (1.95)	6.69 (2.38)	< 0.001
Friendliness	7.54 (1.86)	6.87 (2.23)	< 0.001
Aptitude	7.44 (1.99)	6.72 (2.36)	< 0.001
Empathy	7.36 (2.01)	6.71 (2.36)	< 0.001

**Figure 2:**
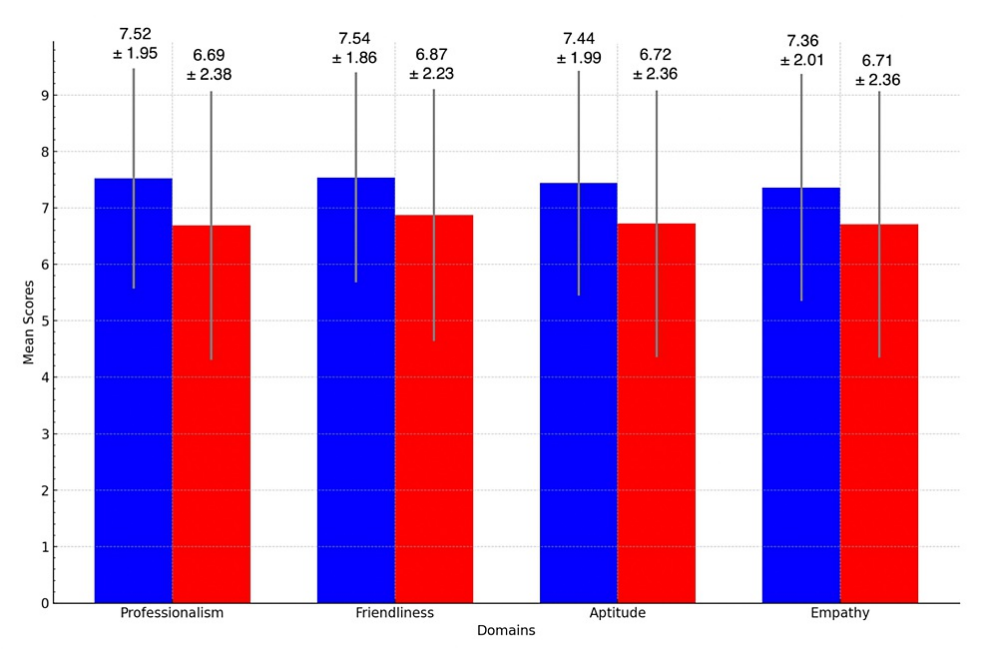
Patient ratings of Formal Office Attire vs. Scrubs (Mean with standard deviation) Blue: scrubs; Red: formal office attire.

Patients rated doctors who were wearing the scrubs as more professional [mean 7.52 (SD 1.95) vs. 6.69 (SD 2.38), p< 0.001], friendlier [mean 7.54 (SD 1.86) vs. 6.87 (SD 2.23), p< 0.001], with higher aptitude [mean 7.44 (SD 1.99) vs. 6.72 (SD 2.36), p < 0.001] and more empathetic [mean 7.36 (SD 2.01) vs. 6.71 (SD 2.36), p < 0.001]. There was no difference in opinion between the inpatients and outpatients. When analyzed by age of patients, there was no significant difference in opinion in both young and old patients. Both groups of patients rated scrubs to be significantly more favorable in all four domains (professionalism, friendliness, aptitude, and empathy) (Figure 2).

Patient ratings of formal office attire vs. scrubs (gender subgroup analysis)

Scrubs were statistically superior to formal office attire in all four domains for both female and male physicians. For male physicians, scrubs were rated more professional [mean 7.55 (SD 1.93) vs. 6.85 (SD 2.31), p< 0.001], friendlier [mean 7.56 (SD 1.82) vs. 6.93 (SD 2.20), p < 0.001], with higher aptitude [mean 7.45 (SD 1.98) vs. 6.82 (SD 2.29), p < 0.001] and more empathetic [mean 7.38 (SD 2.00) vs. 6.80 (SD 2.28), p < 0.001].

**Figure 3:**
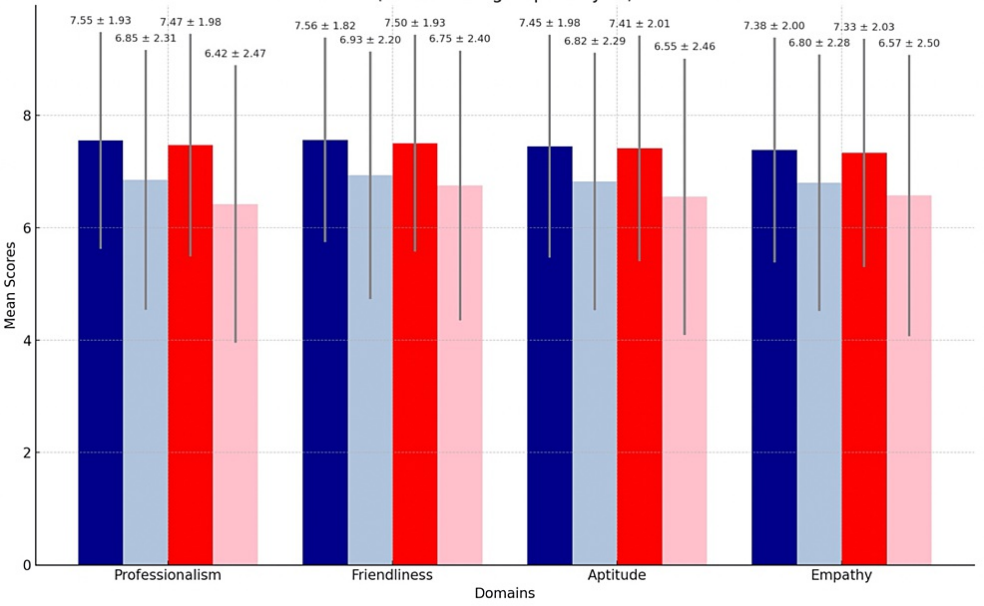
Patient ratings of Formal Office Attire vs. Scrubs (Gender Subgroup Analysis) (Mean with standard deviation) Dark Blue: male scrubs; Light Blue: male formal office attire: Red: female scrubs; Pink: female formal office attire

Female physicians scored similarly: professionalism [mean 7.47 (SD 1.98) vs. 6.42 (SD 2.47), p< 0.001], friendliness [mean 7.50 (SD 1.93) vs. 6.75 (SD 2.40), p < 0.001], aptitude [mean 7.41 (SD 2.01) vs. 6.55 (SD 2.46), p < 0.001], empathy [mean 7.33 (SD 2.03) vs. 6.57 (SD 2.50), p < 0.001] (Figure 3).

When analyzing male vs female physicians, female physicians scored poorer in professionalism [mean 6.95 (SD 2.30) vs. 7.20 (SD 2.16), p < 0.01] compared to male physicians. There was no difference in the other domains (Table 4).

**Table 4:** Domain scores between Female vs. Male physicians

Domain	Scrubs Mean (SD)	Formal Office Attire Mean (SD)	P-Value
Professionalism	6.95 (2.30)	7.20 (2.16)	< 0.01
Friendliness	7.13 (2.21)	7.24 (2.05)	0.14
Aptitude	6.98 (2.30)	7.13 (2.16)	0.06
Empathy	6.95 (2.21)	7.09 (2.17)	0.07

When assessed by the different attires, we found that for formal office attire, Female physicians scored significantly poorer in professionalism as compared to their male counterparts [mean 6.42 (SD 2.47) vs. 6.85 (SD 2.31), p < 0.001] (Table 5).

**Table 5:** Domain scores between Female vs. Male physicians in Formal Office Attire

Domain	Females Mean (SD)	Males Mean (SD)	P-Value
Professionalism	6.42 (2.47)	6.85 (2.31)	< 0.001
Friendliness	6.76 (2.40)	6.93 (2.20)	0.14
Aptitude	6.55 (2.46)	6.82 (2.30)	0.03
Empathy	6.57 (2.50)	6.80 (2.28)	0.06

All other domains did not exhibit statistically significant differences. However, for scrubs, females scored comparably to males in all four domains (Table 6).

**Table 6:** Domain scores between Female vs. Male physicians in Scrubs

Domain	Females Mean (SD)	Males Mean (SD)	P-Value
Professionalism	7.47 (1.98)	7.55 (1.93)	0.44
Friendliness	7.50 (1.93)	7.56 (1.82)	0.54
Aptitude	7.41 (2.01)	7.45 (1.98)	0.71
Empathy	7.33 (2.03)	7.38 (2.00)	0.59

Patient ratings of formal office attire vs. scrubs (age of physician subgroup analysis)

Whilst younger doctors tended to score higher in friendliness and professionalism, there was no significant difference in all four domains between young and old doctors (Table 7).

**Table 7:** Domain scores between Young vs. Old physicians

Domain	Young Physicians Mean (SD)	Old Physicians Mean (SD)	P-Value
Professionalism	7.17 (2.18)	7.04 (2.25)	0.09
Friendliness	7.27 (2.07)	7.13 (2.15)	0.06
Aptitude	7.12 (2.19)	7.03 (2.23)	0.20
Empathy	7.09 (2.20)	6.98 (2.24)	0.18

## Discussion

In this study, we examined the effect of doctors' attire on patients' perceptions of professionalism and other related domains of friendliness, aptitude, and empathy. Scrubs and formal office attire were chosen as they are the most commonly donned amongst local physicians. This study found that patients overwhelmingly preferred doctors in scrubs over those in formal office attire. Despite patients themselves stating that a doctor’s attire had no bearing on their perception, this was found to be untrue. These findings challenge the conventional notion that formal office attire is a primary indicator of professionalism in the medical field. Importantly, the preference for scrubs was a viewpoint shared between both younger and older patients. This preference for physicians in scrubs suggests a possible shift in the way patients perceive professionalism in healthcare.

Physician attire is steeped in culture and tradition [12]. White coats have long been synonymous with physicians since medicine’s shift from pseudoscience to science in the 20th century, and, much like the stethoscope, is a visual identifier in clinics today [13]. It also portrays an image of cleanliness and professionalism that may confer trust and authority [13]. In our local context, white coats are not often donned. Doctors in our local hospitals often dress in formal office attire or scrubs, without the addition of a white coat. Indeed, it is notable that patients did not highlight the white coat as a sign of being a doctor. Instead, patients readily accepted that doctors often wear Scrubs, with subtle differentiating factors such as color, to distinguish them from nursing staff and allied health associates.

The findings from our study differed greatly from other large-scale studies in other centers, which showed a clear preference for formal office attire over scrubs [10,11,14-17]. It would seem that our local patients do not perceive formal office attire to reflect aptitude and professionalism amongst physicians, and in fact, may find that formal office attire reflects less friendliness and poorer empathy. Several factors may contribute to this difference. In the local healthcare context, scrubs are often donned by physicians in various sub-specialties. This was particularly true during the COVID-19 pandemic, where scrubs were encouraged due to a stringent focus on infection control. Even after the pandemic, several healthcare workers have shown a preference for routine scrub attire [18]. Repeated exposure to physicians in scrubs, may have bred a sense of familiarity amongst patients that has resulted in expecting physicians to be dressed in scrubs rather than the formal office attire. Attire familiarity as a factor is comforting [19], and may explain why patients view scrubs favorably in terms of friendliness and empathy. Secondly, the local cultural milieu may also play a role. Geographic variation and differing cultures have been shown to affect patients’ preference for physician attire [17]. In our local tropical climate, it can be uncomfortable to wear layered clothing and as such, patients may recognize that wearing scrubs is comfortable and practical for the physician. As such, there is minimal effect on perceived professionalism and aptitude. Some studies have found that the public prefers physicians in sub-specialties such as emergency medicine and surgery to be donned in scrubs [17]. In this study, patients under the care of varying sub-specialties were interviewed and a sub-group analysis of the patients did not reveal any preferences with regard to sub-specialties. 

In this study, most patients felt that the color of scrubs would be a defining factor between physicians and nursing/allied health staff. They were, however, unable to articulate the color difference. In addition, patients also highlighted the importance of name tags in differentiating physicians from nursing/allied health staff. As such, scrub color schemes and prominent nametags should be presented to patients. This study was not designed to determine patient perceptions based on physician gender. Nonetheless, it was noted that female physicians were rated poorer than males in professionalism. This is consistent with several published data [20-22]. In terms of attire, it was noted that this difference was accentuated when females in formal office attire were compared to males in Formal Office Attire. The differences disappeared in Scrubs. Given our findings, the authors recommend that the ideal healthcare attire should be Scrubs, with a uniform color scheme and a prominent name tag.

Limitations

This study was conducted in a controlled environment to minimize bias from confounding factors. One limitation of this study was the method by which we presented our patients with their options. Our study utilized controlled photographs, which limited our patients’ ability to identify the differences in the attires based on dynamic cues. In practice, attires are not perceived in isolation based on static images. A future study could potentially utilize short-form videos that show physicians in their separate attires, with controls for body language and facial expressions to minimize bias. As part of an extension to this limitation, Female physician attires were not standardized in this study. Thus, it was not possible to determine if a standardized Female office attire would reduce perception differences between Male and Female physicians as shown in this study. Secondly, we did not review other attires such as white coats, as this attire is now infrequently worn locally. Nonetheless, a study that included other less common attires such as White Coats could give us a better idea of patient preferences amongst a larger range of attires. Finally, our study was also limited in its focus on 4 domains of perception - professionalism, friendliness, aptitude, and empathy. A future study could also analyze the difference in perceptions of important factors of the patient experience, such as bedside manner, quality of counseling, and quality of care.

## Conclusions

This study highlights that physician attire can affect how patients view physicians. Whilst the majority of patients feel that physician attire would not affect their perceived professionalism, it was evident that physician attire did have an effect. Patients view physicians in scrubs as having higher professionalism, friendliness, aptitude, and empathy as compared to physicians in formal office ttire. We recommend that physicians should don standardized colored scrubs with a prominent name tag to improve patient perceptions. 
